# A fast and efficient algorithm for DNA sequence similarity identification

**DOI:** 10.1007/s40747-022-00846-y

**Published:** 2022-08-23

**Authors:** Machbah Uddin, Mohammad Khairul Islam, Md. Rakib Hassan, Farah Jahan, Joong Hwan Baek

**Affiliations:** 1 Department of Computer Science and Engineering, University of Chittagong, Chittagong, 4331 Bangladesh; 2 Department of Computer Science and Mathematics, Bangladesh Agricultural University, Mymensingh, 2202 Bangladesh; 3 School of Electronics, Telecommunication and Computer Engineering, Korea Aerospace University, Goyang-si, South Korea

**Keywords:** DNA sequence similarity, Dynamic *k* - $k-mer$, Matrix shrinking, AFproject, Benchmark dataset, Bioinformatics engineering

## Abstract

DNA sequence similarity analysis is necessary for enormous purposes including genome analysis, extracting biological information, finding the evolutionary relationship of species. There are two types of sequence analysis which are alignment-based (AB) and alignment-free (AF). AB is effective for small homologous sequences but becomes *NP*-hard problem for long sequences. However, AF algorithms can solve the major limitations of AB. But most of the existing AF methods show high time complexity and memory consumption, less precision, and less performance on benchmark datasets. To minimize these limitations, we develop an AF algorithm using a 2D $k-mer$ count matrix inspired by the CGR approach. Then we shrink the matrix by analyzing the neighbors and then measure similarities using the best combinations of pairwise distance (PD) and phylogenetic tree methods. We also dynamically choose the value of *k* for $k-mer$. We develop an efficient system for finding the positions of $k-mer$ in the count matrix. We apply our system in six different datasets. We achieve the top rank for two benchmark datasets from AFproject, 100% accuracy for two datasets (16 S Ribosomal, 18 Eutherian), and achieve a milestone for time complexity and memory consumption in comparison to the existing study datasets (HEV, HIV-1). Therefore, the comparative results of the benchmark datasets and existing studies demonstrate that our method is highly effective, efficient, and accurate. Thus, our method can be used with the top level of authenticity for DNA sequence similarity measurement.

## Introduction

Sequence analysis is a trending research arena in the field of bioinformatics, bioinformatics engineering, and computation biology. It is obligatory for analyzing the evolutionary relationship among different living objects from whole genomes, finding gene regulatory regions, identifying virus–host interactions, detecting horizontal gene transfer, analyzing the similarity of sequences, extracting different biological information, etc. [28]. Day by day, biological information extraction from the whole genome is becoming important because of rapid expansion (approximate growth rate is doubling data in every 18 months) of biological data from the last few decades [34]. Broadly, there are two types of sequence analysis: AB and AF where AB algorithms have several limitations. For example, it provides better results only for homologous sequences, it works for comparatively smaller sequences and these algorithms are time and space consuming. For multiple and long sequences, it becomes *NP*-hard problem. However, AF algorithms can solve the major limitations of the AB algorithms [40, 41]. Due to their time and memory efficiency, AF methods are widely used in different free, paid, open and publicly available software including MEGA (Molecular Evolutionary Genetics Analysis) [13], MEGA7/X [13], CAFE (aCcelerated Alignment-FrEe sequence analysis) [22], Co-Phylog [35], etc.

Different AF-based researches have been conducted on sequence similarity analysis. Among them, pattern histogram [23], suffix tree count [18], $k-mer$ encoding-based image analysis [6–8], chaos game representation (CGR) approach [2, 30, 38], convolutional neural network (CNN) approach using CGR image [30] are extensively used in different studies. We discuss different AF models, their strengths and limitations in the second section. From that analysis, we find that most of the AF-based approaches have some general limitations, e.g., high time complexity, high memory consumption rate, less precision, lack of optimal $k-mer$ selection, achieving high performance by testing their model in smaller datasets, and lack of comparison to benchmark dataset.

Therefore, in this research, we aim to develop an AF sequence similarity measurement model that will overcome the limitations of existing models. For any sequence, our model dynamically selects *k* for $k-mer$ by considering the whole dataset. Then it generates a 2D count matrix of $k-mer$s in a fast and efficient way by utilizing an accurate calculation of the position of $k-mer$ strings in the 2D matrix. After that, it shrinks the 2D matrix by analyzing neighbors and then generating a 1D feature descriptor. Then, we experiment to find the best combinations of distance and phylogenetic tree generation methods to achieve high precision. Thus, the method effectively calculates the similarities of any sequence dataset.

The rest of the manuscript is organized as follows. In the next section, we discuss different existing AF models with their strengths and limitations. In the subsequent section, we present our novel sequence similarity measurement method. Then we discuss different datasets, performance on different datasets, and performance of the overall system in comparison to existing studies. Finally, we summarize the contributions and limitations of our system. Also, we put some future directions.

The details of the dataset, implemented code are publicly available (https://drive.google.com/drive/folders/1NIJUqtHryV7nhzPRbKyJT8U6ZTYpre2U?usp=sharing).

## Background study

Ren et al. [28] performed a comparative study to analyze the pros and cons of different AF algorithms in the field of sequence analysis. The study also mentioned that the AB approaches provide higher accuracy than AF methods. However, AF methods are computationally efficient and have less memory consumption rate. Yang et al. [34] mentioned different approaches in their study for encoding DNA sequence to numbers e.g., sequential, one-hot [33] and $k-mer$ encoding. They presented several issues, e.g., choosing appropriate encoding, feature extraction technique, choosing the right distance measuring technique may affect overall performance.

Jin et al. [14] analyzed different methods used for DNA sequence similarity identification and they mentioned that a good similarity algorithm should have the following ability (i) should have a strong encoding technique to reduce the information loss, (ii) extracted features should work for small, large and mixed length (the length varies from $$10^2 $$ to $$10^{10}$$ or more) sequence, (iii) should have high precision, less time complexity, and space consumption rate. Luczak et al. [23] surveyed to evaluate different histogram-based distance matrices used for phylogeny analysis. They mentioned that to achieve better accuracy, the size of $$k-mer$$ should be increased for comparatively larger sequences. Zielezinski et al. [40] developed a benchmark for comparing thousands of AF algorithms developed by targeting different sequence analysis studies. They launched a web portal named AFproject^1^ in which anyone can submit their self developed AF algorithm to evaluate the comparative performance score among reference algorithms and datasets. Klötzl et al. [18] developed a suffix tree based algorithm and claimed their method is faster and accurate than Mash [26] and other pairwise algorithms. However, in the AFproject web portal, they obtained RF distance of 6.00 for fish dataset.

Chen et al. [7] developed a method for phylogeny analysis where they converted a DNA sequence to a digital vector by assigning $1-mer$ ($A=1, C=2, G=3,\text { and }T=4$) and combined it with index information. After that, a gray level co-occurrence matrix (GLCM) was calculated from the vector. Again, Chen et al. [6] extended their previous work using $2-mer$ and got comparatively good results in respect to previous studies. However, in both studies, the dataset was very small in comparison to the benchmark dataset. Similarly, Somodevilla et al. [32] used $1-mer$ ($A=1, C=0.5, G=0.75,\text { and }T=1$) encoding for generating an image. Later, they used CNN for DNA sequence classification. However, they faced a time complexity issue. Delibaş et al. [8] proposed a method by utilizing first-order statistical concepts from an image texture. They used four small datasets and compared their dendrogram with MEGA7, and ClustalW. However, they did not apply their method for a large benchmark dataset to find their methods’ accuracy, error and rank. Again, Delibaş et al. [9] proposed $top-kn-gram$ based solution and calculated $top-kn-gram$ from the count of $k-mer$s. They applied their system in different datasets including AFproject [40] fish benchmark dataset where they achieved rank 6 with 68% accuracy.

Chaos game representation (CGR) is a square matrix of $k-mer$ counts in a genome sequence. Traditionally, it is calculated based on coordinate values of ancestor and predecessor DNA bases [2, 30]. Zheng et al. [38] used the CGR image technique for circRNA disease association finding. Dick et al. [10] mentioned that coordinate point-based CGR calculations have several limitations. For that reason, they proposed four different CGR (FCGR, 20-node-amino-acid CGR, 20-node-amino-acid FCGR, and 20-Flake-FCGR) representations for protein classification. However, Changchuan Yin [36] showed that coordinate-based CGR matrix calculation highly suffers from floating-point error and an integer representation may provide a good result. Safoury et al. [30] worked for DNA sequence classification using convolutional neural network (CNN) from CGR image. They prepared a square matrix of $k-mer$ sequences, where each cell contains the number of counts of a specific sequence and their accuracy was 51% to 100%. In addition, the method takes a huge time to generate split images to train CNN. Rizzo et al. [29] developed a DNA sequence classification based on CGR image. Löchel et al. [20] also used deep learning (DL) techniques for proteins classification and used CGR images to train the DL model. All of the studies mentioned that DL-based methods have huge time complexity. Kania et al. [17] analyzed the behavior of CGR implementations and sequence correlations and found that there was a strong relationship between $k-mer$ with accuracy. Besides, $k-mer$ frequency counting CGR (FCGR) methods were more sensitive for representing mutations, but it increased the time and space complexity.

Ni et al. [25] developed a method for DNA sequence similarity where they used the FCGR technique. Generally, $8-mer$ generates a 2D matrix of $(4^4 \times 4^4)$ dimension that contains $4^8=65,536$ pixels. They reduced this vector with the concept of bicubic interpolation technique which returns a 2D matrix of $(16 \times 16)$ dimension, then it was converted to 1D $(1 \times 256)$ vector. Thus, it reduces the vector size as well as time complexity. Later, perceptual image hashing difference was used for sequence similarity calculation. This method was tested on 21 HIV-1, 48 HEV, 8 mammalian chromosome DNA, and 25 Fish DNA from AFproject [40]. Among the results, for AFproject [40] dataset they obtained rank 2, RF distance 4, and score 91. Hence, the method reduces the time complexity and achieved a good performance. However, for the same dataset other methods exist in AFproject [40] that achieve performance rank 1, RF distance 2, and accuracy 95. So, there are scopes to improve the accuracy or optimize the time complexity and accuracy.

Based on the rigorous literature review, we summarize the general limitations of existing systems:

- AB algorithms are accurate but not suitable (*NP*-hard) for larger sequences. Because AB algorithms rearrange sequence bases based on blocks or segments, and the number of rearrangements becomes very large with increasing sequence length and the number of input sequences.
- Different AF algorithms are available. Some of the methods demonstrated good results but takes more time to execute, while other methods take less time but provide relatively worse results. Hence, the development of an efficient and effective AF algorithm is a crucial need for not compromising between accuracy and time.
- Performance of the $k-mer$-based AF methods highly depends on the number of *k*. Existing studies show that the accuracy of the method increases for choosing larger *k*, but the space and time consumption also increases with *k* which indicates less performance. However, there is no effective algorithm to select the optimal value of *k* for $k-mer$ dynamically.
- Most of the existing AF algorithms suffer from high time complexity and memory space consumption problem. Practically, the time and memory required to measure sequence similarity in existing methods are very high. However, biological researchers expect a method with less time and memory requirements.
- Traditional coordinate-based implementation of CGR may be unable to represent the mutations or slight changes in sequences. In addition, these techniques suffer from floating-point errors. Although FCGR algorithms can represent mutations, they have high time complexity. Hence, a new CGR algorithm is needed in which sensitivity and time complexity are optimized.
- Extracting a very few numbers of features from CGR image degrades the accuracy. Again, a large number of feature extraction from CGR images increases the distance calculation time. Hence, an optimal number of feature extraction from a CGR image is a very challenging task.
- Applying a model on a small dataset having short sequences may provide a good result, but it may not be true for the benchmark dataset. For practical use, a method having good results should be tested on benchmark datasets.
- A method should be tested in an open platform like AFproject [40] web portal so that the researchers and users can publicly see the performance. It will help to compare a new model.

Hence, we aim to develop a DNA sequence similarity technique that will address all of the above limitations.

## Proposed methodology

**Fig. 1:**
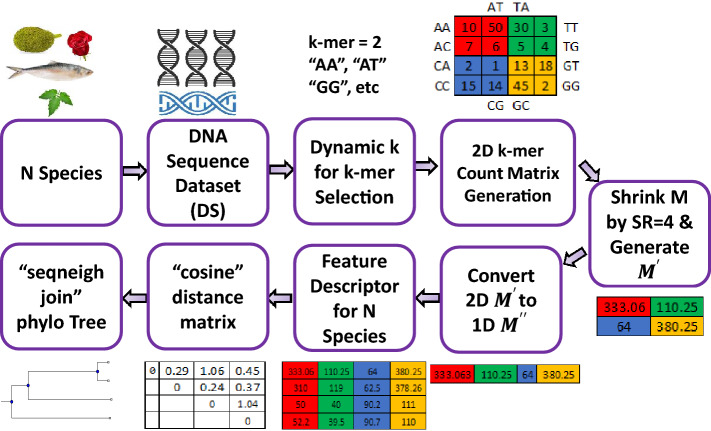
Overview of our proposed DNA sequence similarity identification model

In this section, we describe the detailed procedure of our proposed sequence similarity identification from raw sequences. The overall procedure of our proposed method is presented in Fig. 1.

### Dynamic *k* for $k-mer$ selection

In DNA sequence analysis research, the AF algorithm works better than AB algorithms [28]. Generally, genome sequences (e.g., “AATTTTTAACG”) are large string consisted of different DNA bases (“A”,“T”,“C”,“G”). AB algorithm considers whole string and aligns one by one base, hence, these algorithms show huge time complexity for analyzing multiple sequences. On the contrary, the AF algorithm considers different smaller DNA sequence subsets which are known as $k-mer$ and then applies different count, histogram, network or probability algorithms. A $k-mer$ is a subset of a DNA sequence of a specific length [23]. However, AF algorithms represent the large sequences by a different form, e.g., number rather than a string. Different researchers used various lengths of subsets in their AF model. Safoury et al. [30] used two different values of *k* for $k-mer$ which is very time consuming. However, the performance of a model highly depends on choosing the right number of *k* [14, 23]. As it has a crucial role, choosing an appropriate number of *k* is very challenging and developing a method to choose the dynamic number of *k* is time demanding [23].

Therefore, we propose Algorithm 1 for finding the appropriate number of *k* for $k-mer$. In this algorithm, first, we read *N* number of sequences from the dataset, then make a vector *V* to keep the individual lengths of *N* sequences, then the average length *L* is calculated from *V*. Based on *L*, the algorithm selects the value of *k*.
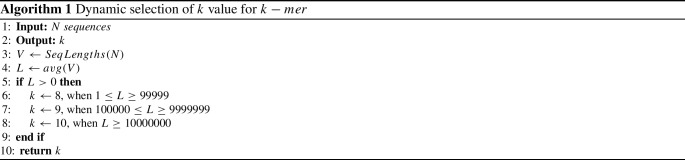


### DNA sequence to 2D $k-mer$ count matrix

After choosing an appropriate number of *k* using Algorithm 1, our process generates a 2D matrix using Algorithm 2 which is graphically presented in Fig. 1. Different researchers used a coordinate-based CGR approach to generate a 2D matrix from a sequence where they used a coordinate averaging technique to move from the previous point to the next point [2, 10, 30, 38]. However, due to averaging technique, these methods have suffered from floating-point error which interrupts achieving high precision. Moreover, different studies used frequency chaos game representation (FCGR) for the sequence to image conversion. Adetiba et al. [1] developed an FCGR from the derivatives of CGR images where they found improved accuracy for increasing the number of derivatives. But the derivative process is computationally inefficient. Löchel et al. [20] developed a FCGR matrix based on contraction ratio which is suffers from floating-point error. Joseph et al. [16] also used the FCGR technique by dividing the CGR image into 4 blocks where each block was generated by averaging the coordinates of base points. This method also suffers from floating-point error. Rizzo et al. [29] used deep learning-based FCGR image analysis where they calculated the frequency of a $k-mer$ by iterating the whole sequence. So, their method consumes a huge amount of time to execute. Messaoudi et al. [24] implemented a technique for FCGR calculation by moving a template window ($k-mer$) among the whole sequence and counting the number of full matches. They generate a different number of orders of FCGR to enhance the performance. This method also consumes huge CPU time which is almost $O(n^2)$. Different studies [11, 19, 25, 39] developed FCGR matrix based on coordinate averaging technique for detecting biological sequence. Therefore, it is necessary to develop an accurate and time-efficient CGR count matrix for sequence analysis.

**Fig. 2:**
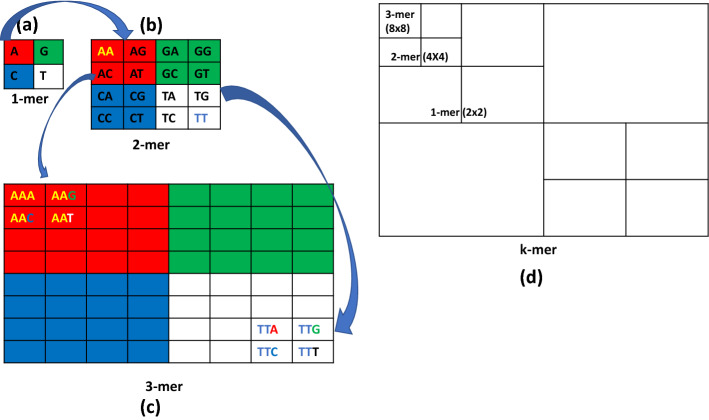
Two-dimensional $k-mer$ count matrix where each cell represent a number of count of a specific subset in the whole string **a** $1-mer$ has 4 count cells for the subset with one base, **b** $2-mer$ contains 16 count cells for subset comprising of two bases, **c** $3-mer$ has 64 count cells where each subset is comprised of 3 bases or a codon, **d** general expansion formula for *k* length subset or $k-mer$. Here, four red color cells in (**b**) indicate that it is expanded from one red cell in (**a**), again, 16 red cells in (**c**) indicate that it is expanded from red cells of (**b**)


Hence, we aim to develop a method that will generate a 2D $k-mer$ count matrix where the cells of the matrix are distributed according to CGR formation. CGR is a method that iteratively represents the bases (“A”,“T”,“C”,“G”) of a DNA sequence using the coordinates of a square matrix *M* or gray level image where the size of matrix is ($2^k \times 2^k$), here *k* is the length of $k-mer$ string. This process assigns one cell for each $k-mer$ string using Algorithm 3, and the value of each cell indicates the frequency of the specific $k-mer$ string using Algorithm 2. It is possible to reconstruct the source sequence from the coordinates by backtracking. This CGR square matrix or gray level image is suitable for finding the similarities among DNA sequences [2, 25]. Our method calculates the position of a $k-mer$ string in the 2D matrix using Algorithm 3 without averaging technique. Hence, 2D count matrix generation by our method will be highly effective in terms of accuracy and time. In Fig. 2, we present the 2D matrix expansion process. Therefore, using Algorithm 3 and Algorithm 2 we generate a 2D count matrix.

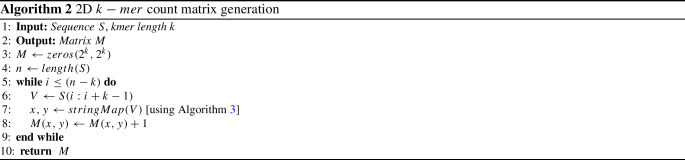

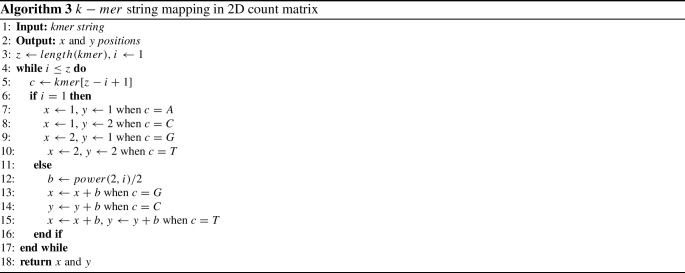


### Matrix shrinking and feature descriptor

A $k-mer$ count matrix contains the major detailed information of a sequence. With the increase of $k-mer$, the size of the count matrix also increases significantly. If we use the whole matrix as a feature vector, it will be computationally ineffective. But, it is necessary to develop a method that will work for long, medium and short length sequences [14]. So, different researchers proposed different methods (e.g., linear interpolation, bicubic interpolation [25]) for matrix or data shrinking. In the 2D $k-mer$ matrix, each cell is important as it contains the number of occurrences of a specific sequence in the whole sequence. Ni et al. [25] used bi-cubic interpolation which shrinks the vector enormously but the performance of their method on the benchmark dataset is not very promising. Hence, we propose a method to shrink the $k-mer$ count matrix that calculates the square of the mean value of neighboring elements. The detailed procedure is presented in Algorithm 4.

Hence, according to Algorithm 4 for a known *k* or $k-mer$, the dimension of the count matrix *M* is $(d \times d)$ where $d=4^{k/2}$ and *k* is length of $k-mer$. If we shrink the vector by shrink rate $S_r$ then the output matrix $M_s$ will be $(d^\prime \times d^\prime )$ where $d^\prime =d/sqrt(S_r))$. Then, convert 2D $M_s$ to 1D $D_s'$ by row column shifting. Hence, $D_s'$ is the feature descriptor.
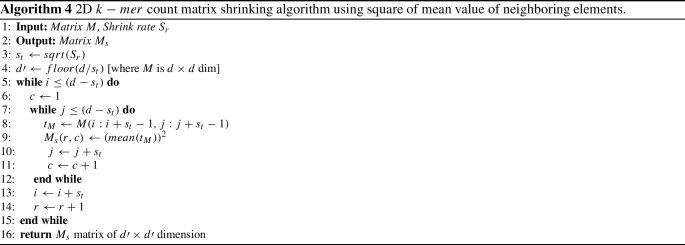


### Cosine distance and phylogenetic tree construction

Statistical distance calculation methods highly depend on data and pattern distributions [23]. Cosine similarity provides good results for $k-mer$ probabilities or the count matrix [5, 23, 27]. Let the length of descriptor *D* be the dimension of the vector. It calculates the angle between two vectors using Eq. 2. The smaller value of angle indicates a good similarity which also indicates the two vectors are parallel. However, cosine distance is measured by $1-cosine$ value.

Let, the two descriptors for two sequences are $x=D_{s1}$ and $y=D_{s2}$ and their cosine is the inner product of two vectors divided by their magnitude defined in Eq. 1.

1 $$\begin{aligned} Cos(\theta )= \frac{\mathbf {x}.\mathbf {y}}{\Vert \mathbf {x}\Vert \Vert \mathbf {y}\Vert } = \frac{\sum _{k=1}^{n} x_k . y_k}{\sqrt{\sum _{k=1}^{n} x_k^2}\sqrt{\sum _{k=1}^{n} y_k^2}}, \end{aligned}$$

where *n* is the length of descriptor *x* and *y*, here both descriptors are of same length. The upper part of the equation represent the dot product of the vectors and $\Vert \mathbf {x}\Vert$ and $\Vert \mathbf {y}\Vert$ represent the magnitude of the vector *x* and *y* respectively. Again, $x_k$ is the $k^{th}$ element of descriptor *x* and $y_k$ is the $k^{th}$ element of descriptor *y*.

2 $$\begin{aligned} L= 1-Cos(\theta ), \end{aligned}$$

where $Cos(\theta )$ is measured in Eq. 1.

Hence, the *L* value is the cosine distance between two sequences. Therefore, we can apply this technique for more than two sequences by adopting the one-to-one comparison technique. If the number of sequences is *n*, then the length of *L* will be $z=(n * (n-1))/2$, thus the dimension will be $1 \times z$, respectively.

Again, a phylogenetic tree is a prime tool to visualize the genetic relationship [3]. We use *seqneighjoin* function which takes a new value from distance matrix *L* that is considered as a new node *q*. Then it computes the distance of *q* versus all existing nodes. Hence, in each iteration, a new *q* is considered and overall similarity values are updated. For any node *q* the distance matrix calculation is presented in Eq. 3.

3 $$\begin{aligned} L(q,p)= & {} x*L(i,p) + (1-x)*L(j,p) \nonumber \\&- x*L(q,i) - (1-x)*L(q,j), \end{aligned}$$

where *L* is the distance matrix, *q* is a new node, $i \text { and }j$ are the iteration variable, *p* is the set of all existing nodes, *x* is 1/2 for *eqiuvar* [31], and $0 \text { to }1$ for *firstorder* [12] method.

**Table 1:** Description of 25 cichlid fish genome sequences

SL	Description	Accession	Seq. Length
1	Tropheus duboisi	009063	16,747
2	Tropheus moorii	018814	16,826
3	Petrochromis trewavasae	018815	16,828
4	Neolamprologus brichardi	009062	16,823
5	Oreochromis aureus	013750	16,867
6	Oreochromis niloticus	013663	16,866
7	Oreochromis sp. KM_2006	009057	16,865
8	Tanganyika Tylochromis polylepis	011171	17,118
9	Hypselecara temporalis	011168	16,782
10	Astronotus ocellatus	009058	16,807
11	Ptychochromoides katria	011169	16,794
12	Paratilapia polleni	011170	16,760
13	Paretroplus maculatus	011177	16,723
14	Etroplus maculatus	011179	16,693
15	Abudefduf vaigiensis	009064	16,943
16	Amphiprion ocellaris	009065	16,888
17	Cymatogaster aggregata	009059	16,771
18	Ditrema temminckii	009060	16,810
19	Pseudolabrus eoethinus	012055	16,745
20	Pseudolabrus sieboldi	009067	16,747
21	Pteragogus flagellifer	010205	17,034
22	Halichoeres melanurus	009066	17,039
23	Parajulis poecilepterus	009459	16,896
24	Alepocephalus agassizii	013564	16,677
25	Bajacalifornia megalops	013577	17,290

**Table 2:** Description of 8 Yersinia strains

SL	Description	Accession	Seq. Length
1	Y. pestis Antiqua	CP000308	4,702,289
2	Y. pestis Nepal516	CP000305	4,534,590
3	Y. pestis F_15-70	NC009381	4,517,345
4	Y. pestis CO92	AL590842	4,653,728
5	Y. pestis KIM	AE009952	4,600,755
6	Y. pestis 91001	AE017042	4,595,065
7	Y. pestis pseudotuberculosis IP32954	BX936398	4,744,671
8	Y. pestis pseudotuberculosis IP31758	AAKT 02000001	4,721,828

## Results and discussion

In this section, we discuss dataset collection, the performance achieved on benchmark and existing datasets, the effectiveness of our model, and some comparison with existing works. We use a total of 6 standard genome datasets that are collected from different benchmarks and existing studies. Among them, we use the first 2 datasets for benchmark testing, the second 2 for comparing the accuracy and the rest 2 for memory and space analysis.

The details of the 6 standard genome datasets are (i) complete mitochondrial DNA sequences of 25 cichild fish samples (Table 1), (ii) 8 Yersinia strains (Table 2), (iii) 16 S ribosomal DNA of 13 Bacteria  [8, 9], (iv) 18 Eutherian mammals  [8, 9], (v) HIV-1 [25], and (vi) HEV [25].

Among them, the first 2 are open challenge datasets from AFproject [40] where they evaluate the performance and ranking of different AF algorithms used for sequence similarity identification. Rest 2 (16 S ribosomal, 18 Eutherian mammals) are collected from different existing works [8, 9]. We use another 2 datasets (HIV-1, HEV) for memory and space analysis those are taken from Ni et al. [25] which can be found from the following URL.^2^

**Table 3:** *k* and $S_r$ selection using four datasets

Dataset	*k*	RF distance for Different $S_r$				
		$S_r=1$	$S_r=4$	$S_r=16$	$S_r=64$	$S_r=256$
Fish (Table 1)	8	2	2*	8	12	12
	9	2	2	4	6	10
	10	2	2	4	8	12
Yersinia (Table 2)	8	2	2	2	6	10
	9	0	0*	2	2	6
	10	0	0	2	4	6
16 S Ribosomal	8	0	0*	4	6	12
	9	0	0	4	10	16
	10	0	0	4	10	18
18 Eutherian	8	0	0	0*	2	10
	9	0	0	2	6	6
	10	0	0	2	4	10

Note: Column 1 tells about the dataset used in the experiment, column 2 indicates the *k* value used for 2D matrix generation, columns 3–7 show RF distance obtained using different $S_r$. Here, (*) sign indicates the best combinations of *k* and $S_r$

**Table 4:** RF distances for different distance method and phylogenetic tree generation method for Fish dataset in Table 1 using $k=8$ and $S_r=4$

Distance method	Seqlinkage						Seqneighjoin	
	Average	Single	Complete	Weighted	Centroid	Median	Equivar	Firstorder
Euclidean	18	22	20	14	34	34	2*	6
Squaredeuclidean	18	22	20	14	34	32	4	8
Seuclidean	44	44	44	44	44	44	8	8
Cityblock	8	16	8	10	28	26	4	4
Minkowski	18	20	20	14	34	34	2*	6
Chebychev	38	40	38	38	40	40	36	36
Cosine	8	6	16	16	20	18	2*	2*
Correlation	14	20	16	16	32	30	2*	10
Hamming	8	10	10	10	10	30	4	4
Jaccard	8	16	8	8	30	24	4	4
Spearman	6	14	8	8	26	24	4	4

Note: First column indicates the methods used for PD calculation from feature vectors, columns 2–9 represent the RF distance value achieved by differentphylogenetic tree generation techniques: columns 2–7: methods are under *seqlinkage*, columns 8–9: under *seqneighjoin* technique. Here, (*) indicates top result

### Software and server configuration

We simulate our method in 2.80 GHz Intel(R) Core *i*5 computer with 8GB DDR3 RAM. As a development tool, we use the MATLAB 2021a version. The details of the dataset and implemented code are publicly available (https://drive.google.com/drive/folders/1NIJUqtHryV7nhzPRbKyJT8U6ZTYpre2U?usp=sharing).

**Table 5:** RF distances for different distance methods and phylogenetic tree generation methods for Yersinia dataset in Table 2 using $k=9$ and $S_r=4$

Distance method	Seqlinkage						Seqneighjoin	
	Average	Single	Complete	Weighted	Centroid	Median	Equivar	Firstorder
Euclidean	6	4	6	6	8	8	2	2
Squaredeuclidean	6	4	6	6	6	6	0*	0*
Seuclidean	10	10	10	10	10	10	0*	0*
Cityblock	6	4	6	6	8	8	2	0*
Minkowski	6	4	6	6	8	8	2	2
Chebychev	10	10	10	10	10	10	10	10
Cosine	6	4	6	6	6	6	0*	0*
Correlation	6	4	6	6	6	6	0*	0*
Hamming	4	4	6	4	8	8	4	4
Jaccard	4	4	6	4	8	8	4	4
Spearman	4	4	6	4	6	6	0*	0*

Note: First column indicate methods used for PD calculation from feature vectors, columns 2–9 represent RF distance achieved by different phylogenetic tree generation techniques. Columns 2–7 show distance for *seqlinkage* technique, columns 8–9 for *seqneighjoin* technique. Here, (*) indicates top results

### *k* for $k-mer$ and shrink rate ($S_r$) selection

In AF algorithms, the right number of *k* selections plays a vital role in achieving the overall performance of a model [14, 23]. However, increasing the number of *k* also exponentially increases time complexity. Again, it is not optimal to use the full $k-mer$ count matrix as a feature vector because it increases the distance calculation time. So, we develop Algorithm 4 to shrink the vector size. Hence, to build an optimal model, we need to choose the best combination of *k* and $S_r$ with respect to different datasets. Here, $S_r=1$ means no shrink. For each *k* and $S_r$, we experiment with different combinations of pairwise distance (PD) and phylogenetic tree generation methods. Hence, we find 88 combinations (details are available in Table 4) and find 88 RF distances. Among them, we consider the minimum RF value which is listed in Table 3. Therefore, the best result will be the minimum RF value achieved for the combination of a smaller number of *k* and a larger number of $S_r$.

From Table 3, we see that for the Fish dataset (Table 1), best result $RF=2$ achieved for $k=8$ and $S_r=4$. In case of Yersinia dataset (Table 2), best $RF=0$ found for $k=9$ and $S_r=4$. In 16 S Ribosomal dataset, best $RF=0$ for $k=8$ and $S_r=4$, and in the 18 Eutherian Mammal dataset, best $RF=0$ for $k=8$ and $S_r=16$. Generally, with the increase of $S_r$ value, the performance degrades for all *k*. Moreover, we found that minimum *k* value 8 provides the best result for three datasets except for Yersinia. In the case of Yersinia, $k=9$ provides the best result. We investigate the reason and find that the average length of the sequences in the dataset plays a crucial role in selecting *k* value. When the average length is less than $10^5$, then *k* value 8 provides the best result. For Yersinia, the best result for *k* is 9 because its average length is $5 \times 10^6$. In the case of $S_r$, all datasets except 18 Eutherian mammals provide the best result for large $S_r=4$ whereas the 18 Eutherian dataset provides the best result for $S_r=16$. Therefore, we set $S_r=4$ for the four datasets.

**Table 6:** Benchmark test result for 25 complete mitochondrial DNA sequences of cichlid fishes dataset in AFproject test platform

Rank	Method	RF	Accuracy
1*	**(4)SR(K)MER_FEM1**	2.00*	95*
1	8KMERHist+LBP	2.00	95
1	AFKS–d2_star	2.00	95
1	AFKS–d2z	2.00	95
1	AFKS–euclidean_z	2.00	95
1	AFKS–n2r	2.00	95

Here, we present top 5 methods among around 100 methods. Bold and (*) sign represents the performance of our method

Based on the RF distance in Table 3, we develop Algorithm 1 to dynamically select the *k* value. Therefore, we can say that our model is suitable for any DNA sequence similarity dataset and our Algorithm 1 is very effective for the length of $k-mer$ selection. Also, Algorithm 4 shrinks the matrix efficiently.

### PD and sequence joining method selection

To calculate the DNA sequence similarity, we need to measure distances using feature vectors. It involves two steps, first finding the PDs from feature vectors and then generating a phylogenetic tree from distances. MATLAB provides different PDs and phylogenetic tree generation methods. Generally, there may be performance variations in choosing different combinations of PD and phylogenetic tree generation methods. Hence, choosing an appropriate combination of both is a great challenge. Here, we use the best combinations of *k* and $S_r$ selected from Experiment 4.2. To find out which combination is best for our model, first, we apply each tree generation method with each distance method for the Fish dataset $k=8$ and $S_r=4$ and calculate their RF distance presented in Table 4. Further, tree generation methods are of two types, e.g., *seqlinkage* and *seqneighjoin*. Hence, we find 88 RF distances for the Fish dataset for 88 different combinations. From Table 4, we see that the minimum RF distance of 2 marked by * sign is achieved by *cosine* distance and *seqneighjoin* with *firstorder* or *equivar* method. However, we also observe that for all PD methods, *seqneighjoin* technique provides better results than *seqlinkage*. For this dataset, our method achieves the best result (RF distance) in 5 combinations. Interestingly, in all cases, the *seqneighjoin* phylogenetic tree method provides the best results. Therefore, the combinations of *cosine* and *seqneighjoin* is the best pair for Fish dataset sequence similarity.

Similarly, we evaluate our method on Yersinia dataset (Table 2) with $k=9$ and $S_r=4$ in Experiment 4.2. The result is presented in Table 5. This time, we obtain best result RF distance of 0 for 11 different combinations. Five PD methods (*cosine*, *squaredeuclidean*, *seuclidean*, *correlation*, and *spearman*) are combined with *seqneighjoin* which provide good results. However, for both Fish and Yersinia datasets, the combination of *cosine* and *seqneighjoin* provides the top score. Hence, after rigorous experiment on two datasets for 176 combinations, we select *cosine* and *seqneighjoin* methods as the best combination which can be very effective for sequence similarity analysis.

### Performance evaluation on fish benchmark dataset

**Fig. 3:**
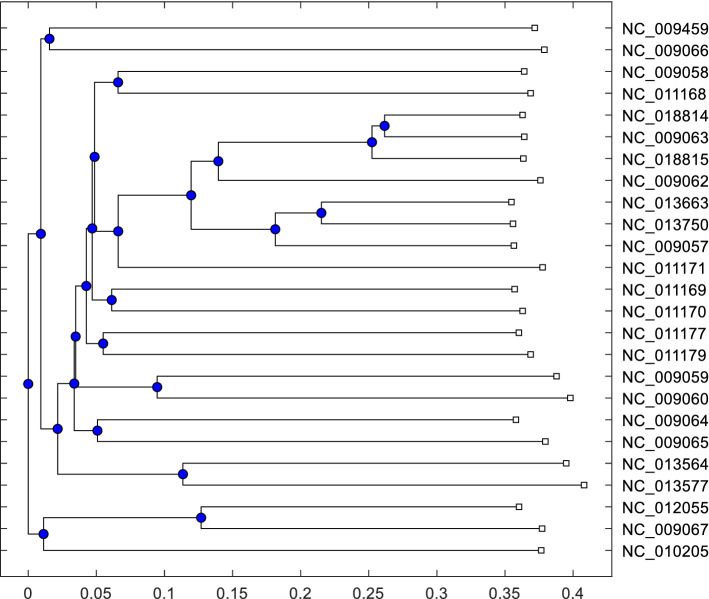
Phylogenetic tree of 25 fish genome sequences described in Table 1. using our proposed method with $k-mer=8$ and $S_r=4$

To evaluate the strength of our proposed algorithm we apply our method in the Fish (Table 1) dataset from AFproject [40]. About one hundred algorithms were submitted for benchmark ranking in the fish dataset. There are 25 sequences for the cichlid genome and their length varies from 16 to 17 thousand bases. These sequences are very similar. Therefore, it is very challenging to identify the accurate similarity or hierarchy for this dataset. AFproject [40] considers three parameters for evaluating algorithms. These are (i) Robinson–Foulds (RF) distance [4, 21] to calculate the distance among phylogenetic trees, (ii) normalized Robinson–Foulds (nRF) that calculates a topological mismatch for a given tree with respect to a reference tree and (iii) normalized quartet distance (nQD). We can convert nRF value to accuracy using Eq. 4.

4 $$\begin{aligned} A= (1-n_{RF}) * 100, \end{aligned}$$

where $n_{RF}$ is normalized Robinson–Foulds value.

To compare the performance among the methods we consider three parameters from AFproject [40] (URL^3^. In Table 6, we list the top 5 methods where our model is on the top rank with RF distance 2.0 and accuracy 95%. Also, in Fig. 3, we present the phylogenetic tree generated by our method. We use $k=8$ for matrix generation and $S_r=4$ for shrinking matrix. The comparative results and phylogenetic tree indicate that our method provides the best result for sequence similarity identification. Besides, Ni et al. [25] applied $k=8$ for $k-mer$ CGR matrix with a dimensionality reduction technique on the same dataset and they achieved rank 2 with RF distance 4.0 and accuracy 91%. However, among 25 sequences 4 sequences are highly similar to one another, due to the reason none of the AF algorithms can achieve 100% accuracy for this dataset. This clearly demonstrates that our method is one of the top-performing methods.

**Table 7:** Benchmark test result for 8 Yersinia strains dataset in AFproject test platform

Rank	Method	RF	Accuracy
1*	**(4)SR(K)MER_FEM1**	0.00*	100*
1	3 M-S64-(K)Mer	0.00	100
1	AFKS–canberra	0.00	100
1	AFKS–chi_squared	0.00	100
1	AFKS–d2_star	0.00	100
1	AFKS–d2s	0.00	100

Here, we present top 5 methods among 80 methods. Bold and (*) sign represents the performance our method

**Fig. 4:**
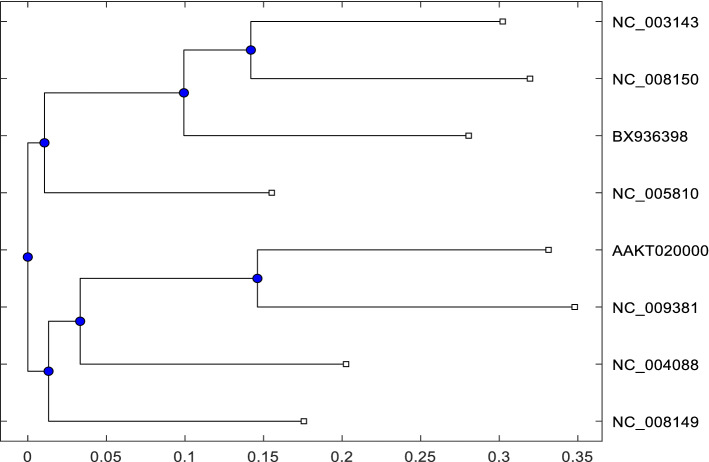
Phylogenetic tree of 8 Yersinia genome sequences described in Table 2 using our proposed method with $k=9$ and $S_r=4$

**Fig. 5:**
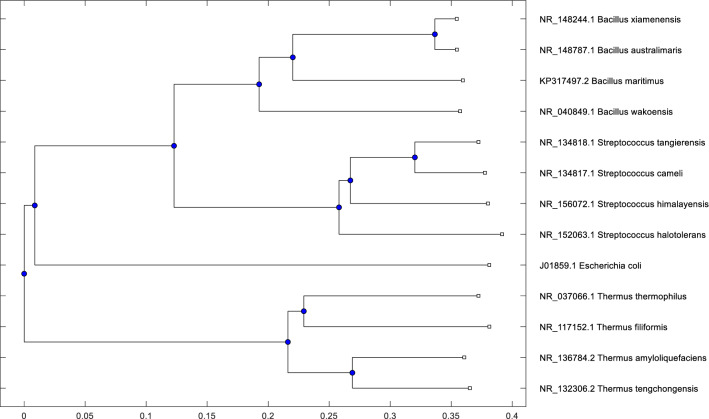
Phylogenetic tree of 16 S Ribosomal DNA sequences of 13 bacteria using our proposed method with $k=8$ and $S_r=4$

### Performance evaluation on Yersinia benchmark dataset

Again, we apply our method in Yersinia (Table 2) benchmark dataset from AFproject [40]. It consists of 8 sequences of Yersinia species where the length varies from 4.5 to 4.7 million bases. However, this dataset is practically large. Approximately 80 algorithms have been submitted for benchmark ranking in the Yersinia dataset. We conduct experiment on similar way of Experiment 4.4 AFproject [40] which can be found on (URL^4^. In Table 7, we list the top 5 methods where our method scores top rank with RF distance 0.00 and accuracy 100%. We also present the similarity identification result using a phylogenetic tree in Fig. 4. Based on Experiment 4.2, to achieve the best result, we use $k=9$ and $S_r=4$ for 2D matrix generation and shrinking. However, according to Table 7, our method achieves the best result for this large dataset which is also inferred in the phylogenetic tree. Hence, it indicates that our method is the best fit for sequence similarity identification. Again, Ni et al. [25] mentioned that if the size of the descriptor is large then it keeps more information than the smaller descriptor. Hence, our solution achieves top ranking among almost 80 algorithms which clearly demonstrates that our method is very suitable for similarity identification from a large sequence dataset.

### Phylogenetic analysis of 16 S ribosomal DNA of 13 bacteria

We choose another dataset from Delibaş et al. [9] which consists of 13 bacterial data of 16 S Ribosomal DNA sequences with description, accession code to access from NCBI URL^5^, and sequence length. Each sequence has a length of approximately 1500 bases. Among the sequences, some of the sequences are highly similar, and the rest are well separated.

First, we generate a Newick tree using *MEGA7/X* software with the following setup: *ClustalW* alignment with default settings of pairwise and multiple alignments. Then we use *UPGMA* mega tree to build the phylogenetic tree and Newick tree string. Second, we generate phylogenetic tree using our proposed method with $k=8$ and $S_r=4$ which are chosen from Experiment 4.2. The phylogenetic tree generated by our method is shown in Fig. 5. Then, we compare the Newick tree generated by *MEGA* and our method, and the comparative result is presented in Table 8. We can see that our method achieves 100% accuracy for this dataset which is very promising and definitely ahead of Delibaş et al. [9] result. It also indicates our method is very effective for the sequence similarity identification of smaller sequences (e.g., 16 S Ribosomal DNA of 13 bacteria).

**Fig. 6:**
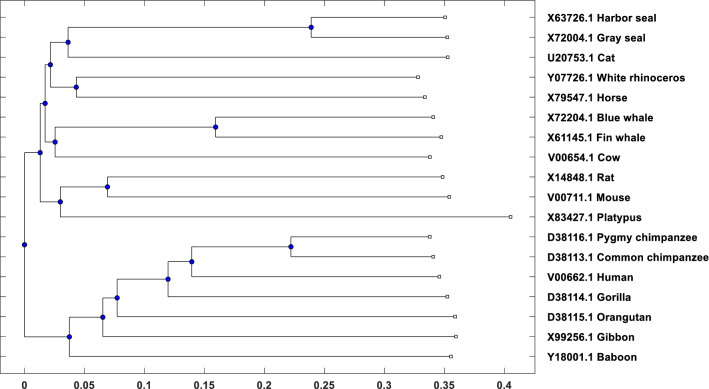
Phylogenetic tree of 18 Eutherian mammals using our proposed method with $k=8$ and $S_r=4$

### Phylogenetic analysis of 18 Eutherian mammals

We choose another existing dataset 18 Eutherian Mammal used by Delibaş et al. [9], Jin et al. [15] etc. Sequence length varies approximately from 16 to 17 thousand. In this dataset, we experiment in two steps like 16 S Ribosomal dataset Experiment 4.6. We generate a phylogenetic tree using $k=8$ and $S_r=4$ is shown in Fig. 6 and then compare the Newick tree with *MEGA7*. The comparative result is presented in Table 9. We can see that our method achieves 100% accuracy for this dataset too which is very promising and clearly ahead (19% more accurate) from Delibaş et al. [9] result. It also indicates our method is very effective and efficient for whole genome DNA sequence similarity identification.

### Performance in terms of time and space

The effectiveness of any computer algorithm is measured by several parameters. Among them, the time complexity is most important [37]. Because it indicates how faster an algorithm can provide results. Different researchers including Delibaş et al. [9] computed the complexity in terms of machine clock cycle. However, we discuss our time complexity in two steps. First, we express time complexity using $\theta$ and *O* notation. Let, the sequence dataset consists of *N* number of sequences, where each sequence has a maximum of *L* length and *k* is the length of $k-mer$ string. There are several steps to compute the time complexity which are presented in Table 10. Hence, total complexity is $O(N \times L\times 2^k)$. Second, we calculate the time using *tic* and *toc* function. We also compare the results with existing work in Table 11. Further, space is another parameter that can express the quality of the developed algorithm. We have calculated memory consumption using *memory* function. To compare the memory consumption of our method, we consider HIV-1 and HEV datasets.

From Table 11, we can see that in case of HEV dataset our method is 7,079 times faster than Ni et al. [25]. An almost similar result is obtained for the HEV dataset. Again, our method is approximately 21 times faster than Delibaş et al. [9] for 18 Eutherian mammal dataset. In terms of memory consumption, our method takes 12.85MB less memory than Ni et al. [25] for the HIV-1 dataset. Therefore, we can say that to provide faster results with less memory consumption, our method is the best fit among all existing methods.

**Table 8:** DNA similarity identification accuracy comparison for 16 S Ribosomal DNA dataset

Method	Param 1	Param 2	Accuracy
Proposed method	$k=8$	$S_r=4$	100*
Delibaş et al. [9]	$n- gram=4$	$top-k=15$	91

Here, Column 1 represents the methods, Columns 2 and 3 list most important two parameters, and last column represents the performance achieved by each method. Bold and (*) sign indicates the best result

### Impact of proposed shrinking algorithm

In our system, most time and space consumption part is the count matrix and the next is the pairwise distance calculation. Let, a vector *F* with the dimension of ($N \times P$), where *N* is the number of sequences and *P* is the length of the 1*D* descriptor which is termed as $D_s'$ in “Matrix shrinking and feature descriptor” section. Hence, the computational complexity of pairwise distance calculation is $\frac{N(N-1)}{2} \times 3P$ [23]. Therefore, in our case, the computation highly depends on the value of *P* as *N* is very small compared to *P*. That is why we aim to reduce the size of *P*. In Table 12, we compare phylogenetic tree generation time using different shrink rates and without shrink for all datasets. For all cases, we generate a 2D $k-mer$ count matrix based on the *k* value of the second column, then we calculate the required time using *tic* and *toc* time functions. In this table, we see that with the increase of $S_r$ rate for all datasets the required time is decreasing. When we do not use any shrinking, then the algorithm consumes the most CPU time. It is also observed that if we increase the $S_r$ rate, our method consumes less time. Therefore, we can say that number of features (*P*) in the matrix plays an important role in time consumption and obviously our proposed shrinking algorithm has a considerable impact on overall performance.

**Table 9:** DNA Similarity identification accuracy comparison for 18 Eutherian mammals mitochondrial DNA dataset

Method	Param 1	Param 2	Accuracy
Proposed method	$k=8$	$S_r=4$	100*
Delibaş et al. [9]	$n-gram=13$	$top-k=4$	81

Here, Column 1 represents the methods, Columns 2 and 3 list most important two parameters in each method, and the last column represents the performance achieved by each method. Bold and (*) sign indicates the best result

**Table 10:** Step-wise time complexity calculation for our proposed method

Step	Method	Time complexity
Step 1	Dynamic $k-mer$ selection	$N+2$ (Algorithm 1)
Step 2	2D $k-mer$ matrix generation	$L \times k \times 6$
Step 3	Matrix shrinking	$2^k \times 2^k$
Step 4	1D feature descriptor	$2^k$
Step 5	Distance and phylogenetic tree	$2\times N$
	Final complexity	$O(N \times L\times 2^k)$

**Table 11:** Time complexity and memory space consumption comparison with existing works

Dataset	Method	Time in seconds	Memory in MB
16 S Ribosomal	Our proposed	0.092079*	9.0742
	Delibaş et al. [9]	0.1461	–
18 Eutherian	Our proposed	0.741836*	16.0156
	Delibaş et al. [9]	16.2565	–
HIV-1	Our proposed	0.541657*	16.1836*
	Ni et al. [25]	3600.00	208.00
HEV	Our proposed	1.101700*	24.7422*
	Ni et al. [25]	7200.00	205.00
Fish	Our proposed	1.032676	16.0469
Yersinia	Our proposed	81.321820	75.0531

Column 1 represents name of dataset, Column 2 expresses the method applied on dataset, Column 3 indicates time consumption and Column 4 shows memory consumption. Here, Bold and (*) indicates comparative best result

**Table 12:** Impact analysis of proposed shrinking algorithm in terms of time complexity

Dataset	k-mer value	Required time without shrinking	Required time with shrinking			
			Sr=4	Sr=16	Sr=64	Sr=256
16 S Ribosomal	8	0.145714	0.092079	0.007845	0.002434	0.000945
18 Eutherian	8	1.765345	0.741836	0.069044	0.021210	0.008604
HIV-1	8	1.284576	0.541657	0.012567	0.009631	0.003608
HEV	8	11.65471	1.101700	0.123601	0.087569	0.014063
Fish	8	10.63547	1.032676	0.102035	0.060645	0.010249
Yersinia	9	963.6387	81.32182	9.320472	4.403627	0.990544

## Conclusion

In this research, we develop a method for sequence similarity measurement of any sequence dataset that dynamically selects *k* for $k-mer$ and effectively generates a 2D $k-mer$ count matrix with appropriate shrinking and then applies the best combinations of PD and phylogenetic tree generation method. After comprehensive experiments, we can conclude that our dynamic *k* for $k-mer$ selection algorithm is very essential to achieving the best result. After rigorous experiments on benchmark datasets, comparison with existing studies, phylogenetic analysis and RF distances from reference trees, we can conclude that our 2D $k-mer$ count matrix generation is very much faster, accurate, effective and robust for DNA sequence analysis. Our matrix shrinking, effective position calculation, and optimal combination of PD and phylogenetic tree generation method selection achieve the best performance in terms of time and space. Hence, we can conclude that for sequence similarity analysis our method is novel, robust, faster and accurate. Therefore, we can use it with a good level of reliability.

The contributions of our method are as follows:

- We achieve a top rank score in two benchmark datasets (Fish and Yersinia) among two hundred methods.
- We achieve 100% accuracy for two other datasets (18 Eutherian, 16 s Ribosomal) which are clearly better than other existing methods.
- Our proposed method is faster than existing AF-based methods as well as AB algorithms.
- Proposed system consumes several times less memory than existing methods.
- Our method dynamically choose the value of *k* to generate 2D $k-mer$ matrix using Algorithm 1.
- It takes less time to generate 2D kmer matrix in comparison to others because of our Algorithms 2 and  3.
- Our smart system automatically shrinks the size of feature vector using Algorithm 4 resulting in higher accuracy and minimizing time complexity.

However, our method achieves extraordinary performance for six datasets. In the future, researchers can use more benchmark datasets including COVID 19 and others. Moreover, time and space consumption rates are still a major concern. Finally, researchers can investigate deep learning-based text processing techniques and rough set algorithms for improved performance.

## Footnotes

1 http://afproject.org/app/.

2 https://gitee.com/chinamax2007/fcgr-pih-alignment-free/.

3 http://afproject.org/app/benchmark/genome/std/assembled/fish_mito/results/.

4 http://afproject.org/app/benchmark/genome/hgt/unsimulated/yersinia/results/.

5 https://www.ncbi.nlm.nih.gov/genome/.
